# Comparison of non-coding RNAs in human and canine cancer

**DOI:** 10.3389/fgene.2013.00046

**Published:** 2013-04-08

**Authors:** Siegfried Wagner, Saskia Willenbrock, Ingo Nolte, Hugo Murua Escobar

**Affiliations:** Small Animal Clinic, University of Veterinary Medicine HannoverHannover, Germany

**Keywords:** RNAi, PTGS, model organism, cancer research, dog, miRNA, siRNA

## Abstract

The discovery of the post-transcriptional gene silencing (PTGS) by small non-protein-coding RNAs is considered as a major breakthrough in biology. In the last decade we just started to realize the biologic function and complexity of gene regulation by small non-coding RNAs. PTGS is a conserved phenomenon which was observed in various species such as fungi, worms, plants, and mammals. Micro RNAs (miRNA) and small interfering RNAs (siRNAs) are two gene silencing mediators constituting an evolutionary conserved class of non-coding RNAs regulating many biological processes in eukaryotes. As this small RNAs appear to regulate gene expression at translational and transcriptional level it is not surprising that during the last decade many human diseases among them Alzheimer's disease, cardiovascular diseases, and various cancer types were associated with deregulated miRNA expression. Consequently small RNAs are considered to hold big promises as therapeutic agents. However, despite of the enormous therapeutic potential many questions remain unanswered. A major critical point, when evaluating novel therapeutic approaches, is the transfer of *in vitro* settings to an *in vivo* model. Classical animal models rely on the laboratory kept animals under artificial conditions and often missing an intact immune system. Model organisms with spontaneously occurring tumors as e.g., dogs provide the possibility to evaluate therapeutic agents under the surveillance of an in intact immune system and thereby providing an authentic tumor reacting scenario. Considering the genomic similarity between canines and humans and the advantages of the dog as cancer model system for human neoplasias the analyses of the complex role of small RNAs in canine tumor development could be of major value for both species. Herein we discuss comparatively the role of miRNAs in human and canine cancer development and highlight the potential and advantages of the model organism dog for tumor research.

## Biogenesis and function of small RNAs in mammals

In 2006, Andrew Fire and Craig C. Mello were awarded with the Nobel Prize in medicine for their work on RNA interference (RNAi). Since the discovery of post-transcriptional gene silencing (PTGS) mechanism in various species, the interest in using small RNA molecules and its endogenous mechanisms as a new pharmacological approach to human diseases is constantly rising (Fire et al., 1998; Elbashir et al., 2001). Micro RNAs (miRNA) and small interfering RNAs (siRNA) are two small non-protein-coding RNA molecule types which play a leading role in PTGS. Thereby, contrary to siRNAs, miRNAs appear to act as “fine tuner” of gene regulation (Sevignani et al., 2006).

miRNAs are endogenously expressed as primary miRNA (pri-miRNA) transcripts composed of up to several thousand nucleotides (Mondol and Pasquinelli, 2012) which are processed by the nuclear enzyme Drosha to precursor miRNAs (pre-miRNA) (Zeng and Cullen, 2006). Following the enzymatic processing, the cytoplasmic enzyme Dicer cleaves the pre-miRNAs generating the mature double-stranded miRNAs (Bohnsack et al., 2004; Lund et al., 2004). Dicer not only processes pre-miRNAs, it also cleaves long double stranded RNA molecules and generates the second class of small RNAs, named siRNAs, which show a miRNA-similar size of ~20 base pairs (Tomari and Zamore, 2005).

Mature miRNAs and siRNAs are chemically and physiologically indistinguishable, apparently only differing in their respective origins (Ambros et al., 2003). Further comparison of these molecules shows that the “guide strand” of miRNAs seen in mammals, is in most cases significantly but not obligatory completely complementary to the 3′-untranslated region of the respective target mRNA. In the case of siRNAs the “guide strand” shares absolute complementarity to a small region in the target structure. After “guide strand” incorporation into the RNA-induced silencing complex (RISC), the respective target mRNA stability and/or translation are modulated (Tomari and Zamore, 2005). Interestingly many miRNAs, their biogenesis and functions are conserved among several organisms of higher and lower complexity as fungi, worms, *Drosophila*, and mammals confirming the general importance of the PTGS mechanism (Filippov et al., 2000; Pasquinelli et al., 2000; Wu et al., 2000; Fortin et al., 2002; Yan et al., 2003; Han et al., 2004; Ibanez-Ventoso et al., 2008).

miRNA genes itself show to be very versatile, they were described to be polycistronic or monocistronic and occasionally located in intron- as well as exon-regions of protein-coding genes. Some miRNAs are co-expressed with their target-mRNAs as one transcript (Bartel, 2004; Kim et al., 2009). Different miRNAs target a single mRNA, and a single miRNA can also be regulating the expression of many different targets. Additionally, miRNAs were also reported to be able to regulate other miRNAs by direct interactions (Winter et al., 2009; Chen et al., 2011) and in some cases miRNAs were described to be regulated by proteins translated from their respective target mRNA, constituting a regulatory negative feedback loop (Bracken et al., 2008; Rybak et al., 2008).

In general, long double stranded RNA molecules are not common in mammals, suggesting that the RNAi mechanism mediated by siRNAs evolved for defense of viral infections and transposable elements (Obbard et al., 2009).

Despite their different origin these small non-coding RNA molecules have many things in common including the small size, specificity of inhibition, and potency and considering therapeutic applications a diminished risk to induce unspecific effects as immune responses. Due to these properties, these molecule types are considered to be potential key players in the development of next generation therapeutics for treatment of a variety of major diseases including cancer (Barh et al., 2010).

miRNA transcription and maturation is not the only process regulating functional miRNA levels. Stability of functional miRNAs is a further key factor in miRNA regulation. Molecule stability was reported to be dependent on several cis- and trans-acting factors varying considerably between miRNAs and their spatiotemporal expression (Kai and Pasquinelli, 2010). Exosomal release of miRNAs into the extracellular environment (Ohshima et al., 2010) and long non-coding RNAs (lncRNA) mimicking target mRNA sites and thereby acting as decoys, were also shown to decrease functional miRNA levels in cells (Cesana et al., 2011). Interestingly, numerous lncRNAs were reported to be deregulated in human cancer (Shore et al., 2012).

Typically one strand of the mature miRNA hybrid, the “guide strand” is maintained during interaction with RISC proteins while the “passenger strand” is degraded. This dichotomy is generally known to be caused by the stabilization of the “guide strand” by RISC loading, while the “passenger strand” stays unprotected (Kai and Pasquinelli, 2010). miRNA methylation, uridylation, and adenylation are some of the modifications having an influence on small RNAs half-life as well (Burroughs et al., 2010; Kai and Pasquinelli, 2010). However, miRNA stability-enhancing proteins were also described to be actively involved in miRNA half-life, suggesting that the miRNA-mediated gene regulation processes are more complex and as variable as these genes are itself (Kai and Pasquinelli, 2010).

## The dog as model organism

In recent years, the role of the domestic dog as model organism for various human diseases constantly gained increasing importance. Many canine inherited diseases were described, including Alzheimer's disease (Rofina et al., 2003), narcolepsy (Lin et al., 1999), diabetes (Ionut et al., 2008), epilepsy (Lohi et al., 2005), atrial fibrillation (Shan et al., 2009), Duchenne muscular dystrophy (Mizuno et al., 2011), heart diseases (Eaton et al., 1995), and cancer (Mueller et al., 2007; Boggs et al., 2008; Noguchi et al., 2011; Uhl et al., 2011). All these disorders occur in dogs, just as in humans, spontaneously during their lifetime and many of them show similar clinical manifestations (Ostrander et al., 2000; Sutter and Ostrander, 2004).

Cancer is a complex, polygenic disease spontaneously occurring in man and dog (~1 million diagnosed pet dog cancer cases per year in the United States), whereas tumors in most laboratory animals must be artificially induced (Mueller et al., 2007; Karlsson and Lindblad-Toh, 2008; Paoloni and Khanna, 2008). Indeed, mans best friend shares many features, including tumor genetics, molecular targets, histological appearance, and response to conventional therapies (Paoloni and Khanna, 2008). Additionally dogs often cohabitate with their owners, are exposed to similar environmental stresses, which may have a big impact on cancer development, and enjoy the best medical care among pets (Sutter and Ostrander, 2004; Rowell et al., 2011). Furthermore, dogs show a higher genetic variability than inbred laboratory mice and enable an easier and faster surgical intervention and imaging due to body size (Mueller et al., 2007). The five- to eight-fold faster aging of dogs in comparison to humans facilitates long-term studies of cancer treatments (Rowell et al., 2011). In 2005, the sequenced canine genome was published (Lindblad-Toh et al., 2005). Having a less different genome from humans than rodents and sharing a similar metabolism, according to their body size, the dog classifies as a very good model organism for molecular studies on human diseases (Sutter and Ostrander, 2004; Karlsson and Lindblad-Toh, 2008; Pinho et al., 2012).

In contrast to investigations on human miRNAs in cancer, the research on canine miRNAs is often limited by the lack of specific canine assays. Up to now only a limited number of studies were done on canine non-neoplastic and tumor tissues. In 2008, Zhou et al. identified 357 canine miRNA-genes by computational analysis, 300 of these were homologs of known human miRNAs (Zhou et al., 2008). Currently 1527 human and 323 canine miRNA matches of hairpin precursors are registered in the web-database, miRBase (Sanger Institute, version 16.0) (Kozomara and Griffiths-Jones, 2011).

Due to high homology of mature miRNAs between human and dog (Table 1), many of the human miRNA assays can be used for analyses of canine miRNA expression. In Table 1 canine miRNAs are listed, which share absolute homology to the human counterparts. Homologous canine miRNAs with overhangs or major sequential differences are not listed.

**Table 1:** **Comparison of canine and human mature miRNAs**.

**Canine mature miRNA**	**Human mature miRNA**
cfa-miR-1	hsa-miR-1
cfa-let-7a	hsa-let-7a
cfa-let-7b	hsa-let-7b
cfa-let-7c	hsa-let-7c
cfa-let-7e	hsa-let-7e
cfa-let-7f	hsa-let-7f-5p
cfa-let-7g	hsa-let-7g-5p
cfa-miR-7	hsa-miR-7-5p
cfa-miR-9	hsa-miR-9-5p
cfa-miR-10	hsa-miR-10a-5p
cfa-miR-15a	hsa-miR-15a-5p
cfa-miR-15b	hsa-miR-15b-5p
cfa-miR-16	hsa-miR-16-5p
cfa-miR-17	hsa-miR-17-3p
cfa-miR-18a	hsa-miR-18a-5p
cfa-miR-18b	hsa-miR-18b-5p
cfa-miR-19a	hsa-miR-19a-3p
cfa-miR-19b	hsa-miR-19b-3p
cfa-miR-20a	hsa-miR-20a-5p
cfa-miR-20b	hsa-miR-17-5p
cfa-miR-21	hsa-miR-21-5p
cfa-miR-22	hsa-miR-22-3p
cfa-miR-23a	hsa-miR-23a-3p
cfa-miR-23b	hsa-miR-23b-3p
cfa-miR-25	hsa-miR-25-3p
cfa-miR-26a	hsa-miR-26a-5p
cfa-miR-27a	hsa-miR-27a-3p
cfa-miR-27b	hsa-miR-27b-3p
cfa-miR-28	hsa-miR-28-3p
cfa-miR-29a	hsa-miR-29a-3p
cfa-miR-29b	hsa-miR-29b-3p
cfa-miR-29c	hsa-miR-29c-3p
cfa-miR-30b	hsa-miR-30b-5p
cfa-miR-30e	hsa-miR-30e-3p
cfa-miR-33	hsa-miR-33a-5p
cfa-miR-34a	hsa-miR-34a-5p
cfa-miR-34c	hsa-miR-34c-5p
cfa-miR-92a	hsa-miR-92a-3p
cfa-miR-92b	hsa-miR-92b-3p
cfa-miR-93	hsa-miR-93-5p
cfa-miR-95	hsa-miR-95
cfa-miR-96	hsa-miR-96-5p
cfa-miR-98	hsa-miR-98
cfa-miR-99a	hsa-miR-99a-5p
cfa-miR-99b	hsa-miR-99b-5p
cfa-miR-101	hsa-miR-101-3p
cfa-miR-103	hsa-miR-103a-3p
cfa-miR-105a	hsa-miR-105-5p
cfa-miR-106a	hsa-miR-17-5p
cfa-miR-106a	hsa-miR-106a-5p
cfa-miR-106b	hsa-miR-106b-5p
cfa-miR-107	hsa-miR-107
cfa-miR-122	hsa-miR-122-5p
cfa-miR-125a	hsa-miR-125a-5p
cfa-miR-125b	hsa-miR-125b-5p
cfa-miR-126	hsa-miR-126-5p
cfa-miR-127	hsa-miR-127-3p
cfa-miR-128	hsa-miR-128
cfa-miR-129	hsa-miR-129-5p
cfa-miR-130a	hsa-miR-130a-3p
cfa-miR-130b	hsa-miR-130b-3p
cfa-miR-133b	hsa-miR-133b
cfa-miR-133c	hsa-miR-133a
cfa-miR-134	hsa-miR-134
cfa-miR-135a-5p	hsa-miR-135a-5p
cfa-miR-135b	hsa-miR-135b-5p
cfa-miR-136	hsa-miR-136-5p
cfa-miR-137	hsa-miR-137
cfa-miR-138a	hsa-miR-138-5p
cfa-miR-143	hsa-miR-143-3p
cfa-miR-145	hsa-miR-145-5p
cfa-miR-146a	hsa-miR-146a-5p
cfa-miR-146b	hsa-miR-146b-5p
cfa-miR-147	hsa-miR-147b
cfa-miR-148a	hsa-miR-148a-3p
cfa-miR-148b	hsa-miR-148b-3p
cfa-miR-149	hsa-miR-149-5p
cfa-miR-150	hsa-miR-150-5p
cfa-miR-151	hsa-miR-151a-5p
cfa-miR-152	hsa-miR-152
cfa-miR-153	hsa-miR-153
cfa-miR-155	hsa-miR-155-5p
cfa-miR-181a	hsa-miR-181a-5p
cfa-miR-181b	hsa-miR-181b-5p
cfa-miR-181d	hsa-miR-181d
cfa-miR-182	hsa-miR-182-5p
cfa-miR-183	hsa-miR-183-5p
cfa-miR-184	hsa-miR-184
cfa-miR-185	hsa-miR-185-5p
cfa-miR-186	hsa-miR-186-5p
cfa-miR-187	hsa-miR-187-3p
cfa-miR-190a	hsa-miR-190a
cfa-miR-190b	hsa-miR-190b
cfa-miR-191	hsa-miR-191-5p
cfa-miR-192	hsa-miR-192-5p
cfa-miR-193a	hsa-miR-193a-5p
cfa-miR-193b	hsa-miR-193b-5p
cfa-miR-194	hsa-miR-194-5p
cfa-miR-196a	hsa-miR-196a-5p
cfa-miR-197	hsa-miR-197-3p
cfa-miR-199	hsa-miR-199a-3p
cfa-miR-200a	hsa-miR-200a-5p
cfa-miR-200b	hsa-miR-200b-5p
cfa-miR-200c	hsa-miR-200c-3p
cfa-miR-202	hsa-miR-202-5p
cfa-miR-203	hsa-miR-203a
cfa-miR-204	hsa-miR-204-5p
cfa-miR-205	hsa-miR-205-5p
cfa-miR-206	hsa-miR-206
cfa-miR-208a	hsa-miR-208a
cfa-miR-208b	hsa-miR-208b
cfa-miR-212	hsa-miR-212-5p
cfa-miR-214	hsa-miR-214-3p
cfa-miR-216a	hsa-miR-216a-5p
cfa-miR-216b	hsa-miR-216b
cfa-miR-218	hsa-miR-218-5p
cfa-miR-219	hsa-miR-219-5p
cfa-miR-219^*^	hsa-miR-219-2-3p
cfa-miR-221	hsa-miR-221-3p
cfa-miR-222	hsa-miR-222-3p
cfa-miR-223	hsa-miR-223-3p
cfa-miR-299	hsa-mir-299
cfa-miR-301a	hsa-miR-301a-3p
cfa-miR-301b	hsa-miR-301b
cfa-miR-302a	hsa-miR-302a-5p
cfa-miR-302c	hsa-miR-302c-5p
cfa-miR-302d	hsa-miR-302d-5p
cfa-miR-320	hsa-miR-320a
cfa-miR-323	hsa-miR-323a-3p
cfa-miR-324	hsa-miR-324-5p
cfa-miR-326	hsa-miR-326
cfa-miR-328	hsa-miR-328
cfa-miR-329b	hsa-miR-329
cfa-miR-330	hsa-miR-330-5p
cfa-miR-331	hsa-miR-331-3p
cfa-miR-335	hsa-miR-335-5p
cfa-miR-338	hsa-miR-3065-5p
cfa-miR-33b	hsa-miR-33b-5p
cfa-miR-340	hsa-miR-340-5p
cfa-miR-342	hsa-miR-342-3p
cfa-miR-346	hsa-miR-346
cfa-miR-361	hsa-miR-361-5p
cfa-miR-362	hsa-miR-362-5p
cfa-miR-365	hsa-miR-365a-3p
cfa-miR-367	hsa-miR-367-5p
cfa-miR-370	hsa-miR-370
cfa-miR-374a	hsa-miR-374a-5p
cfa-miR-374b	hsa-miR-374b-5p
cfa-miR-375	hsa-miR-375
cfa-miR-376a	hsa-miR-376a-3p
cfa-miR-376b	hsa-miR-376b-3p
cfa-miR-377	hsa-miR-377-5p
cfa-miR-379	hsa-miR-379-5p
cfa-miR-381	hsa-miR-381-3p
cfa-miR-383	hsa-miR-383
cfa-miR-410	hsa-miR-410
cfa-miR-421	hsa-miR-421
cfa-miR-423a	hsa-miR-423-5p
cfa-miR-424	hsa-miR-424-3p
cfa-miR-425	hsa-miR-425-5p
cfa-miR-432	hsa-miR-432-5p
cfa-miR-433	hsa-miR-433
cfa-miR-448	hsa-miR-448
cfa-miR-449	hsa-miR-449a
cfa-miR-450b	hsa-miR-450b-5p
cfa-miR-451	hsa-miR-451a
cfa-miR-452	hsa-miR-452-5p
cfa-miR-454	hsa-miR-454-3p
cfa-miR-455	hsa-miR-455-5p
cfa-miR-487a	hsa-miR-487a
cfa-miR-487b	hsa-miR-487b
cfa-miR-488	hsa-miR-488-5p
cfa-miR-489	hsa-miR-489
cfa-miR-490	hsa-miR-490-3p
cfa-miR-491	hsa-miR-491-3p
cfa-miR-493	hsa-miR-493-3p
cfa-miR-494	hsa-miR-494
cfa-miR-495	hsa-miR-495-3p
cfa-miR-496	hsa-miR-496
cfa-miR-497	hsa-miR-497-5p
cfa-miR-499	hsa-miR-499a-5p
cfa-miR-500	hsa-miR-500a-3p
cfa-miR-504	hsa-miR-504
cfa-miR-505	hsa-miR-505-5p
cfa-miR-532	hsa-miR-532-5p
cfa-miR-539	hsa-miR-539-5p
cfa-miR-542	hsa-miR-542-3p
cfa-miR-543	hsa-miR-543
cfa-miR-544	hsa-miR-544a
cfa-miR-551a	hsa-miR-551a
cfa-miR-551b	hsa-miR-551b-3p
cfa-miR-568	hsa-miR-568
cfa-miR-574	hsa-miR-574-3p
cfa-miR-590	hsa-miR-590-3p
cfa-miR-599	hsa-miR-599
cfa-miR-628	hsa-miR-628-5p
cfa-miR-652	hsa-miR-652-3p
cfa-miR-660	hsa-miR-660-5p
cfa-miR-671	hsa-miR-671-3p
cfa-miR-708	hsa-miR-708-5p
cfa-miR-758	hsa-miR-758-3p
cfa-miR-761	hsa-miR-761
cfa-miR-802	hsa-miR-802
cfa-miR-874	hsa-miR-874
cfa-miR-875	hsa-miR-875-5p
cfa-miR-876	hsa-miR-876-5p
cfa-miR-885	hsa-miR-885-5p
cfa-miR-1306	hsa-miR-1306-5p
cfa-miR-1307	hsa-miR-1307-3p

*207 of the canine mature miRNAs listed in miRBase (Sanger Institute, version 16.0) show absolute sequence complementarity to the human counterparts. The sequence identity of the canine mature miRNA sequences with the corresponding human homologs was confirmed by miRBase, single sequence search (http://www.mirbase.org/search.shtml)*.

## Comparative miRNA expression in human and canine diseases

Humans share many diseases with their canine companions including atrial fibrillation, Duchenne muscular dystrophy, and cancer, but the number of comparative studies, focussing on the role of miRNAs in canine diseases, is still relatively low (Karlsson and Lindblad-Toh, 2008; Shan et al., 2009; Mizuno et al., 2011). However, the published data is constantly growing and thus it is likely that in future miRNA studies on the canine model, like in the following examples, will gain more importance.

### miRNAs in non-neoplastic diseases

Recent studies showed that nicotine can induce atrial structural remodeling and increase atrial fibrosis vulnerability in dogs. Shan et al. reported a decreased *miR-133* and *miR-590* expression in smoking individuals with atrial fibrosis and showed that an ectopic over-expression of *miR-133* and *miR-590* resulted in a post-transcriptional suppression of TGF-β 1 and TGF-β RII in cultured canine atrial fibroblasts (Shan et al., 2009).

Another disease affecting man as well as dogs is Duchenne muscular dystrophy. It is a lethal X-chromosome linked disorder caused by mutations in the *dystrophin* gene, which encodes a cytoskeletal protein. Mizuno et al. studied serum miRNA expression in the X-linked muscular dystrophy in Japan dog model (CXMD_J_) and found, as in humans, increased *miR-1*, *miR-133a*, and *miR-206* levels (Cacchiarelli et al., 2010, 2011; Mizuno et al., 2011). The study indicates that serum miRNAs might be a reliable biomarker for muscular dystrophy (Mizuno et al., 2011).

### miRNAs in cancer

Focusing cancer in more detail, deregulated miRNA expression was associated with many human and canine neoplasias (Mueller et al., 2007; Barh et al., 2010; Noguchi et al., 2011; Uhl et al., 2011). As miRNAs are involved in a variety of biological processes as regulation of apoptosis, angiogenesis, cell cycle control, and cell migration it is not surprising that these molecules show an enormous influence on cancer etiology (Bueno and Malumbres, 2011; Donnem et al., 2012; Landskroner-Eiger et al., 2012). For example the human *miR-17-92* cluster coded miRNAs where reported to act tumorigenic, while others such as the *let-7* family members, where reported to be like a coin with two sides, acting in some cases as tumor suppressors or promoting tumor development (Blenkiron and Miska, 2007; Boyerinas et al., 2010; Olive et al., 2010; Ryland et al., 2012).

In cancer, miRNA target sites and miRNA genes itself were found to be directly mutated or their expression deregulated by other factors (Ikeda et al., 2011; Ryland et al., 2012). Due to the complex acting and regulation mechanisms it is very likely that many miRNA deregulations associated with their respective disease are not even identified.

However, despite of the fact that the detailed mechanisms of miRNA action are still under debate, many diagnostic and therapeutic miRNA-based approaches show promising results (Li et al., 2009; Krell et al., 2012).

As in humans, in dogs, many miRNAs are conserved emphasizing the role of the domestic dog as model organism for miRNA in cancer research. It is very likely that these molecules also follow comparable expression patterns and similar function in canine neoplasias. The analysis of miRNA biogenesis and expression pattern could decipher the role of human and canine miRNAs in cancer and enable the design of new therapies based on small RNA delivery.

#### miRNAs in mammary tumors

Mammary tumors are among the most common neoplasias of female dogs, with an estimated lifetime risk for malignant tumors varying from 2 to more than 20%. The risk for malignant mammary tumors in dogs spayed before and after their first estrus, is in comparison to intact dogs 0.05 and 8%. In dogs spayed after their second estrus, the risk rises up to 26% (Withrow and Vail, 2007). Data from a Swedish study, based on 80,000 insured female, mostly sexually intact dogs, reported a rate of 111 mammary tumors (benign and malignant) per 10,000 dog-years risk (Egenvall et al., 2005). The age-standardized incidence rate for human breast cancer estimates 66.4 per 100,000 in more developed areas and is thus the most common cancer (Jemal et al., 2011).

According to a recent study, nine miRNAs, *let-7f*, *miR-15a*, *miR-16*, *miR-17-5p*, *miR-21*, *miR-29b*, *miR-125b*, *miR-155*, and *miR-181b* involved in human mammary cancer, appear to follow the same expression pattern in the canine counterpart. In this study, only the investigated *miR-145* was not shown to be differently expressed comparing non-neoplastic and neoplastic canine tissues (Boggs et al., 2008).

#### miRNAs linked to lymphomas

Besides mammary cancer, canine lymphomas show the highest estimated annual incidence with 13 to 24 cases per 100,000 accounting for up to 24% of all canine neoplasias (Withrow and Vail, 2012). In human, chronic lymphocytic leukemia (CLL) is the most common leukemia in the Western world with an annual incidence of approximately 10,000 new cases in the United States (Calin and Croce, 2009).

In a recent study, the relative expression pattern of 12 canine miRNAs *(cfa-let-7a, cfa-miR-15a, cfa-miR-16, cfa-miR-17-5p, cfa-miR-21, cfa-miR-26b, cfa-miR-29b, cfa-miR-125b, cfa-miR-150, cfa-miR-155, cfa-miR-181a, and cfa-miR-223)* in CLL was analyzed. Due to stable expression between the investigated samples four of the 12 miRNAs *(cfa-let-7a, cfa-miR-17-5p, cfa-miR-26b and cfa-miR-223)* were used as endogenous controls. *miR-15a, miR-16, and miR-181a* were reported to be downregulated in canine and human CLL (Calin et al., 2002; Gioia et al., 2011; Zhu et al., 2012). Four of the investigated miRNAs *(cfa-miR-21, cfa-miR-125b, cfa-miR-150, cfa-miR-155)* were described to be upregulated in human and canine B-CLL as well (Pekarsky et al., 2006; Fulci et al., 2007; Wang et al., 2008; Bousquet et al., 2010; Palamarchuk et al., 2010; Rossi et al., 2010). Only *miR-29b*, which was shown to be downregulated in human B-CLL, was upregulated in canine CLL (Pekarsky et al., 2006; Fulci et al., 2007; Wang et al., 2008; Bousquet et al., 2010; Palamarchuk et al., 2010; Rossi et al., 2010).

It was also observed by Gioa et al. that in comparison to canine B-CLL the *miR-125* expression was significantly downregulated in canine T-CLL. On the basis of the mature miRNA expression ratio between *miR-150*/*miR-125b*, and *miR-150*/*miR-155*, it was reported that it is also possible to distinguish among normal blood, B-CLL and T-CLL samples (Gioia et al., 2011). This illustrates the potential of miRNA expression analyses not solely as tumor marker but as an instrument to distinguish between different but similar cell or cancer types.

#### miRNAs associated with melanomas

Melanomas are very aggressive malignant skin cancers in man and dog (Noguchi et al., 2011). Accounting for 5–7% of canine skin tumors. Tumors of the melanocytes and melanoblasts are relatively common in dogs (Withrow and Vail, 2007; Uhl et al., 2011). In human melanoma cell lines A2058, Mewo, and canine melanoma LMeC cells as well as malignant melanoma tissues the *miR-145*, *miR-203*, and *miR-205* expression was reported to be downregulated. An ectopic expression of each of these miRNAs-induced *in vitro* growth inhibition in A2058, Mewo, and LMeC cells (Noguchi et al., 2011, 2012a,b), indicating their potential for treatment of human and canine malignant melanoma.

#### miRNAs involved in epithelial to mesenchymal transition

Furthermore, aberrant activation of the epithelial to mesenchymal transition (EMT) has been observed to promote invasion and metastasis in several human cancers. The EMT inducers ZEB1 and ZEB2 have been shown to be direct targets of the *miR-200* family (*miR-200a*, *miR-200b*, *miR-200c*, *miR-141*, and *miR-429*) in the human breast cancer cell line MDA-MB-231 and in Madin Darby canine kidney epithelial cells (MDCK). Lost expression of these miRNAs was detected in human metaplastic breast cancer specimens, indicating that downregulation of *miR-200* family members may be an important step in tumor progression (Bracken et al., 2008; Gregory et al., 2008; Adam et al., 2009).

#### miRNAs with prognostic significance in osteosarcoma

Representing 1% of diagnosed cancer cases in the United States, osteosarcoma is one of the most common primary malignancies of human bone in children and adolescents (Mirabello et al., 2009). Estimated at >10,000 cases per year, canine osteosarcoma is relatively common in contrast to humans. Like in man, the canine counterpart also arises spontaneously in dogs and shows similar anatomical and functional biology (Khanna et al., 2006; Sarver et al., 2013). Recently Sarver et al. demonstrated an inverse correlation between human 14q32 cluster miRNA expression and aggressive tumor behavior in human and canine osteosarcoma. The group mapped the 14q32 cluster to the canine genome. The *miR-134* and *miR-544* of the 14q32 cluster, showing 100% homology between both species, were used to examine the expression in canine samples. They showed a lower *miR-134* and *miR-544* expression in canine and human bone tumors in comparison to healthy tissues (Sarver et al., 2013). The expression levels of these two miRNAs could provide prognostic utility in osteosarcoma, a disease that shows conserved features between human and dog (Sarver et al., 2013).

For a better overview the previously described miRNA expression patterns in the different canine and human neoplasias were summarized in the Table 2. The described results should be considered with care as major differences could be present in the comparison depending on species, organism the system of tumor classification, type of miRNA preparation and quantification and the used type of normalization.

**Table 2:** **Overview of the above described miRNAs involved in canine and human cancer**.

**MAMMARY TUMORS**			
cfa-let-7f	↑	hsa-let-7f-5p	↑
cfa-miR-15a	↓	hsa-miR-15a-5p	↓
cfa-miR-16	↓	hsa-miR-16-5p	↓
cfa-miR-17-5p	↓	hsa-miR-17-5p	↓
cfa-miR-21	↑	hsa-miR-21-5p	↑
cfa-miR-29b	↑	hsa-miR-29b-3p	↑
cfa-miR-125b	↓	hsa-miR-125b-5p	↓
cfa-miR-145	=	hsa-miR-145-5p	↓
cfa-miR-155	↓	hsa-miR-155-5p	↓
cfa-miR-181b	↑	hsa-miR-181b-5p	↑
**EMT**			
cfa-miR-141	↓	hsa-miR-141-3p	↓
cfa-miR-200a	↓	hsa-miR-200a-5p	↓
cfa-miR-200b	↓	hsa-miR-200b-5p	↓
cfa-miR-200c	↓	hsa-miR-200c-5p	↓
cfa-miR-429	↓	hsa-miR-429	↓
**B-CLL/T-CLL**			
cfa-miR-15a	↓/↓	hsa-miR-15a-5p	↓/–
cfa-miR-16	↓/↓	hsa-miR-16-5p	↓/–
cfa-miR-21	↑/↑	hsa-miR-21-5p	↑/–
cfa-miR-29b	↑/↑	hsa-miR-29b	↓/–
cfa-miR-125b	↑/↓	hsa-miR-125b	↑/–
cfa-miR-150	↑/↑	hsa-miR-150-5p	↑/–
cfa-miR-155	↑/↑	hsa-miR-155-5p	↑/–
cfa-miR-181a	↓/↓	hsa-miR-181a-5p	↓/–
**MELANOMA**			
cfa-miR-145	↓	hsa-miR-145-5p	↓
cfa-miR-203	↓	hsa-miR-203a	↓
cfa-miR-205	↓	hsa-miR-205-5p	↓
**OSTEOSARCOMA**			
cfa-miR-134	↓	hsa-miR-134	↓
cfa-miR-544	↓	hsa-miR-544a	↓

*In the 1st column are the canine and in the 3rd the human miRNAs listed. In the 2nd and 4th column the relative expression or tendencies in comparison to non-neoplastic samples are presented. “–” indicates that no reports were found for involvement in CLL. “=” means that no differences in expression between tumor and healthy cells were described. “↑” signifies upregulation or “↓” downregulation in comparison to non-neoplastic samples*.

## Future prospects

As the majority of miRNAs involved in human and canine diseases are evolutionary conserved, it is likely that the expression patterns are also similar. Nevertheless homologous miRNAs, showing similar pattern of expression in different species, should be considered with care as it is possible that the functions still deviate. Even individual miRNAs in the same species can show oncogene suppressive functions or act oncogenic (Boggs et al., 2008).

However, some miRNAs were shown to be a potential non-invasive biomarker for different clinically relevant subtypes of human breast cancer (Cortez et al., 2012; Shore et al., 2012). As aberrant miRNA expression is partially postulated to be an early event in human tumorigenesis (Cortez et al., 2012) it is tempting to speculate that specific miRNAs could also be used as prognostic tools (Li et al., 2009; Krell et al., 2012) for canine neoplasias and thus should be further evaluated as novel agents in the future.

Further knowledge of spatiotemporal miRNA expression and their respective targets will allow more specific modulation of target or effector molecule expression by delivery of miRNAs, siRNAs, or similar modified oligonucleotides.

A directed ectopic expression of naturally occurring miRNAs could have the capability to act therapeutically in an organism by replenishing the missing tumor suppressor miRNA and interfering with oncogenic properties of cancer cells. In perspective oncomiRs (cancer-promoting miRNAs) could be suppressed by antagomiRs (chemically engineered oligonucleotides that act as miRNA inhibitors) or functionally inhibited by titering them away with lncRNAs (Cesana et al., 2011). Due to the fact that a single miRNA can act on several targets, a miRNA-based therapy could have significant advantages but also bears the risk to induce unintended side effects. Thus, modifications of gene expression by more stringent artificial miRNAs or siRNAs sharing 100% homology to a single target of interest could lower the risk for off target effects, improve treatment, and reduce unwanted side effects.

However, two major obstacles still remain: intracellular delivery and expression level. The ectopic expressed miRNAs must show a certain expression level to reconstitute the “normal” state of genes and the applied small RNAs must be taken up by cancer cells and be further correctly incorporated into RISC. Until now, multiple delivery strategies such as nanoparticles, liposomes, peptide nucleic acids, and viral vectors have been described to achieve this goal but none of these can be used ubiquitously for different types of neoplasias in different locations (Petrocca and Lieberman, 2010; Pan et al., 2011).

Showing many advantages concerning specificity, potency, number of accessible targets, species cross-reactivity, fast development and the scalability, small RNAs may have an enormous diagnostic and therapeutic potential in cancer treatment (Li et al., 2009; Krell et al., 2012) as single agents or e.g., substituting antibody-based cancer therapies.

Homology between human and canine miRNAs could not only enable to use the dog as model organism, but also the transfer of therapeutic and diagnostic approaches established for humans to canines and vice versa. Further elucidation of miRNA functions and biogenesis will facilitate and improve the design and entry of small RNA therapeutic programs into clinical practice. Until now only a few studies describe miRNA expression in canines. Thus, a systematic profiling of miRNA expression would be of great value.

### Conflict of interest statement

The authors declare that the research was conducted in the absence of any commercial or financial relationships that could be construed as a potential conflict of interest.
